# Quantitative PCR methodology with a volume-based unit for the sophisticated cellular kinetic evaluation of chimeric antigen receptor T cells

**DOI:** 10.1038/s41598-020-74927-8

**Published:** 2020-10-21

**Authors:** Syunsuke Yamamoto, Shin-ichi Matsumoto, Akihiko Goto, Miyuki Ugajin, Miyu Nakayama, Yuu Moriya, Hideki Hirabayashi

**Affiliations:** Drug Metabolism and Pharmacokinetics Research Laboratories, Research, Takeda Pharmaceutical Company Limited, 26-1, Muraoka-Higashi 2-chome, Fujisawa, Kanagawa Japan

**Keywords:** Analytical biochemistry, Cancer immunotherapy, Drug discovery

## Abstract

Although the cellular kinetics of chimeric antigen receptor T (CAR T) cells are expressed in units of copies/μg gDNA, this notation carries the risk of misrepresentation owing to dramatic changes in blood gDNA levels after lymphocyte-depleting chemotherapy and rapid expansion of CAR T cells. Therefore, we aimed to establish a novel qPCR methodology incorporating a spike-in calibration curve that expresses cellular kinetics in units of copies/μL blood, as is the case for conventional pharmacokinetic studies of small molecules and other biologics. Dog gDNA was used as an external control gene. Our methodology enables more accurate evaluation of in vivo CAR T-cell expansion than the conventional approach; the unit “copies/μL blood” is therefore more appropriate for evaluating cellular kinetics than the unit “copies/μg gDNA.” The results of the present study provide new insights into the relationship between cellular kinetics and treatment efficacy, thereby greatly benefiting patients undergoing CAR T-cell therapy.

## Introduction

**Figure 1:**
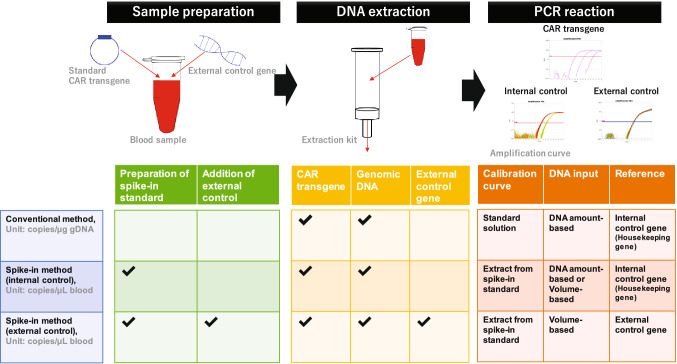
Overview of qPCR methodology. The conventional method expresses the cellular kinetics in units of copies/μg gDNA. DNA from blood samples is extracted, and PCR is performed using a certain DNA input per PCR. The transgene copy number per reaction is calculated using a calibration curve prepared with DNA solution, and normalized to the copy number of an internal control gene, such as a housekeeping gene on gDNA. For the spike-in method, a standard CAR gene is spiked into control blood samples in order to construct the calibration curve. A certain amount of external control gene is added to all samples for the spike-in method with the external control gene. DNA from blood samples is extracted, and PCR is performed. The transgene copy number per volume of blood (copies/μL blood) is calculated using the spike-in calibration curve with normalization based on internal or external control genes.

Several clinical trials of engineered T cells, such as chimeric antigen receptor T (CAR T) cells, have shown that adoptive T-cell therapy is a promising cancer treatment modality^1,2^. Quantitative polymerase chain reaction (qPCR) technique is generally utilized to quantify the administered CAR T cells^3,4^. CAR T cells have a unique blood concentration–time profile: a transient decline a few days after administration and a subsequent rapid in vivo expansion of CAR transgenes^4–7^. The results are expressed in units of transgene copy number per genomic DNA (gDNA) amount (copies/μg gDNA). However, this notation carries a risk of misrepresenting the actual cellular kinetics, as patients generally receive lymphocyte-depleting chemotherapy before CAR T injection. This causes dramatic inter- and intra-individual changes in blood gDNA levels^8,9^. Therefore, we aimed to establish a novel qPCR methodology capable of expressing transgene kinetics based on blood volume (copies/μL blood), as is the case for conventional pharmacokinetic studies of small molecules and other biologics (Fig. 1)^10,11^.

Although DNA extraction efficiency varies across samples^12–14^, transgene extraction efficiency is not taken into account in the conventional method. This is because the ratio of transgene copies to gDNA quantity is constant even if the extraction efficiency is variable. Moreover, PCR is performed using a certain DNA input for each reaction, and the number of transgene copies per reaction is calculated using a calibration curve prepared using a DNA solution, and normalized to the copy number of an internal control gene, such as a housekeeping gene on gDNA^7,15^. Thus, the conventional method enables the quantification of the transgene copy number according to a prescribed amount of gDNA in the DNA eluate.

However, in order to express the results of the cellular kinetics analysis in units of copies/μL blood, the transgene extraction efficiency during DNA extraction should be considered, because the notation indicates the number of transgene copies per a certain volume of blood, rather than that in the DNA eluate. For this method, a standard CAR gene is spiked into control blood samples in order to construct the calibration curve, and is then extracted along with DNA from a certain volume of blood samples. Using this spike-in calibration curve, we can calculate the CAR transgene copy number in blood samples by assuming a constant extraction ratio. However, as DNA extraction efficiency varies across samples^13,14^, it is vital to normalize this variability. Internal control genes are often utilized to normalize the variability in extraction efficiency in the conventional method under conditions of minimal gDNA variability in biological matrices. However, the quantity of DNA per a given volume varies inter- and intra-individually across blood samples as a result of chemotherapy-induced lymphodepletion and inter-individual variability in white blood cell (WBC) counts. Therefore, the variability in extraction efficiency between different blood samples with different background gDNA levels cannot be normalized by cycle threshold (Ct) values of an internal control gene (such as a housekeeping gene). However, this variability can be normalized for the calibration curve or quality control (QC) samples, because pooled blood, with uniform background gDNA content, is utilized to prepare such standard samples.

In this paper, we introduce a novel qPCR method that expresses cellular kinetics in units of copies/μL blood. We utilized the spike-in standard samples for the calibration curve and dog gDNA as an external control to normalize the variability of the extraction ratio, as is done for the conventional LC–MS/MS methods. We evaluate the selectivity, linearity, accuracy, and precision of this method to confirm its quantitative validity. We quantified the CAR transgene level in blood and compared the results expressed in units of copies/μL blood with those expressed in units of copies/μg gDNA, ex vivo and in vivo. Our method enables a more appropriate quantification of the CAR transgene level in blood than the conventional method by eliminating the effect of background gDNA variability. We expect that our qPCR concept will be instrumental in describing “true cellular kinetics” during CAR T-cell therapy.

## Results

### Establishment of qPCR method for volume-based unit

**Figure 2:**
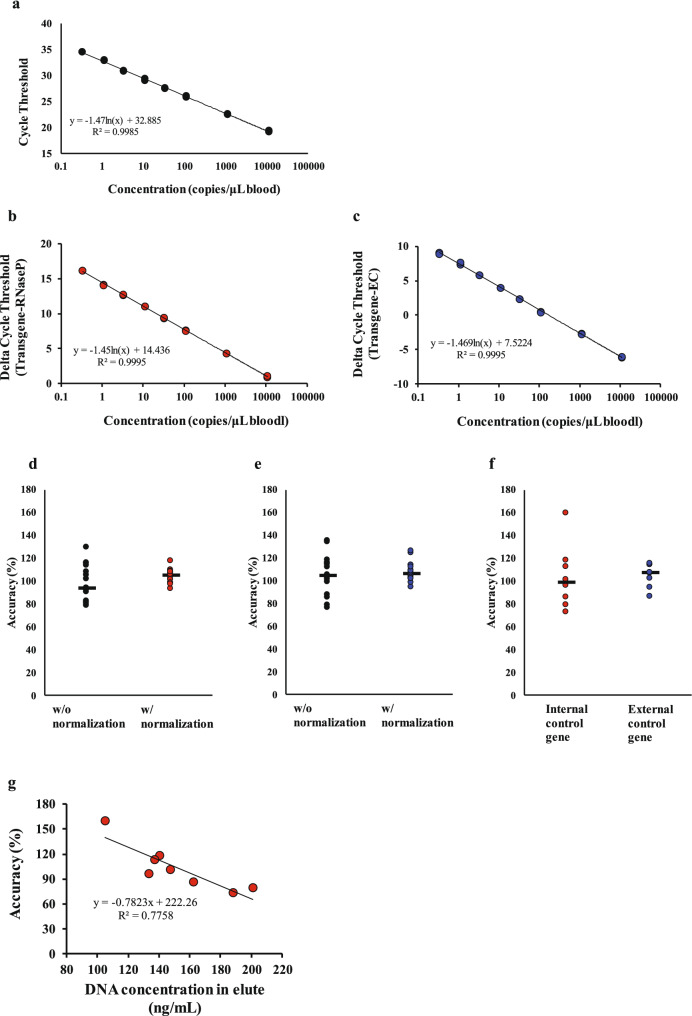
Impact of internal and external control gene on extraction efficiency normalization. Calibration curves plot nominal concentration against cycle threshold for trials with or without normalization (**a**) by internal (**b**) or external control genes (**c**). We compared the accuracy of quality control (QC) samples prepared from pooled blood with or without normalization by an internal (**d**) or external control gene (**e**). The accuracy of QC samples prepared from the blood of eight different donors was assessed for both methods with normalization by internal or external control genes (**f**). The correlation between the DNA concentration in eluate and the accuracy of QC samples prepared from the blood of eight different donors for the method with normalization based on the internal control gene (**g**).

To express the cellular kinetic profile in units of copies/μL blood, transgene extraction efficiency from blood samples was taken into account. We utilized blood samples with a spike-in CAR transgene as the standard sample for the calibration curve in order to incorporate the extraction efficiency into the copy number calculation as described in “Methods” section “Development of qPCR method for volume-based unit,” and Fig. 1. First, we examined the effect of extraction normalization on the linearity of the calibration curve. Linear regression analysis for normalized extraction data showed a greater degree of linearity from 0.3 to 11,111 copies/μL blood (600 to 2 × 10^7^ copies/1.8 mL blood) than for non-normalized data (Fig. 2a), with a coefficient of determination (R^2^) of 0.9995 for both the internal control (human *RNaseP*) and external control (dog *MC1R*; Fig. 2b,c) genes. The PCR efficiencies were acceptable (99.3 and 97.5% for the internal and external control gene, respectively).

Next, we compared the quantitative results of the spike-in method with internal and external control genes using QC samples prepared from pooled blood. We detected no clear difference in the accuracy of the methods, with or without normalization (Fig. 2d,e). However, the coefficients of variation (CV) of two normalized QC samples (5.7% for internal standard and 7.9% for external standard) were lower than those of non-normalized samples (15.3 and 16.6%, respectively; Fig. 2d,e). We then compared the variability of QC samples prepared from the blood from eight different donors with normalization for the conventional and spike-in methods. We found that these samples were more variable than the pooled-blood samples normalized with an internal control gene (CV of 26.7% for QC samples from eight different donors; Fig. 2d,f). The accuracy of QC samples with an internal control gene was inversely correlated with the DNA concentration in the eluate (Fig. 2g). These data suggest that an internal control gene cannot be used to effectively normalize the extraction efficiency from different blood samples, with the varying background gDNA levels. However, it is an applicable method for normalizing the extraction efficiency from a pooled sample, without background gDNA variability. The spike-in method with an external control gene resulted in acceptable accuracy (105.2%) and precision (9.8%) for QC samples (Fig. 2f).

### Validation of spike-in method with external control gene

To validate our method, we prepared the standard and QC samples using pooled human blood and evaluated the linearity, accuracy, and precision on three separate days. As described in Table 1, regression analysis showed high linearity (r^2^ = 0.999 to 1.000) through every data point over the designated concentration ranges of the calibration curve. The slopes of the calibration curves ranged from − 3.39 to − 3.33, suggesting acceptable PCR efficiencies (97.2 to 99.5). The intercepts of the calibration curves were very similar between validation batches. Next, we evaluated the precision and accuracy of the developed method using the QC samples at concentrations of 7.5, 2000, and 20,000 copies/μL blood. The overall accuracy (relative error) and precision (coefficient of variation) were − 9.8 to 9.0% and 4.4 to 10.3%, respectively. These results indicate the robustness of the quantitative method.

**Table 1:** Validation of volume-based qPCR method for transgene detection in human blood.

Nominal concentration (copies/μL blood)	1st day			2nd day			3rd day		
	Mean observed concentration (copies/μL blood)	Relative error (%)	Coefficient of variation (%)	Mean observed concentration (copies/μL blood)	Relative error (%)	Coefficient of variation (%)	Mean observed concentration (copies/μL blood)	Relative error (%)	Coefficient of variation (%)
200,000	218,066.2	9.0	5.4	196,127.5	− 1.9	6.9	190,866.7	− 4.6	9.0
2,000	1895.1	− 5.2	10.3	1803.6	− 9.8	7.4	1852.4	− 7.4	8.1
7.5	8.1	7.4	7.0	8.2	9.4	4.4	7.3	− 2.7	5.7
Slope	− 3.35			− 3.39			− 3.33		
intercept	8.35			8.25			8.37		
PCR efficiency (%)	98.8			97.2			99.5		
R^2^	1.000			0.999			1.000		

We also validated our method using blood from immunodeficient mice. The results from this method, along with those from human blood, provided preclinical validation for the quantitative efficacy of our method (Table 2). Linear regression analysis showed strong linearity (r^2^ = 0.999 to 1.000) and PCR efficiency (93.4 to 95.3%). The y-intercepts of the calibration curve for mouse blood were more variable (12.61 to 14.16) than those for human blood (8.25 to 8.37), which indicates that the extraction efficiencies for mouse blood differed between batches. This could be due to a lower extraction efficiency from immunodeficient mouse blood samples; however, it is notable that we observed acceptable accuracy (relative error: − 23.0 to 20.3%) and precision (coefficient of variation: 2.4 to 19.8%), even under conditions of reduced extraction efficiency. These results reveal that the normalization of extraction efficiency by an external control gene would be an effective method for obtaining quantitatively accurate data in preclinical studies.

**Table 2:** Validation of volume-based qPCR method for transgene detection in mouse blood.

Nominal concentration (copies/μL blood)	1st day			2nd day			3rd day		
	Mean observed concentration (copies/μL blood)	Relative error (%)	Coefficient of variation (%)	Mean observed concentration (copies/μL blood)	Relative error (%)	Coefficient of variation (%)	Mean observed concentration (copies/μL blood)	Relative error (%)	Coefficient of variation (%)
2,000,000	1,832,000	− 8.4	8.8	1,834,000	− 8.3	3.7	1,746,000	− 12.7	4.8
400,000	360,600	− 9.8	4.9	308,000	− 23.0	12.4	332,000	− 17.0	7.9
40,000	35,240	− 11.9	6.1	32,080	− 19.8	7.5	34,540	− 13.6	2.4
4000	4086	2.2	4.0	3712	− 7.2	6.4	3510	− 12.2	3.3
600	665	10.8	8.4	573	− 4.6	10.4	633	5.6	7.2
200	233	16.5	3.8	210	4.8	10.3	184	− 8.0	10.2
100	117	16.8	13.5	112	12.1	18.1	108	8.2	10.3
50	60	20.3	16.9	53	6.6	19.8	52	3.6	17.5
Slope	− 3.49			− 3.44			− 3.44		
Intercept	14.16			12.71			12.61		
PCR efficiency (%)	93.4			95.3			95.2		
R^2^	0.998			0.999			0.999		

### Impact of unit notation on dose-proportionality of transgenes in human blood

**Figure 3:**
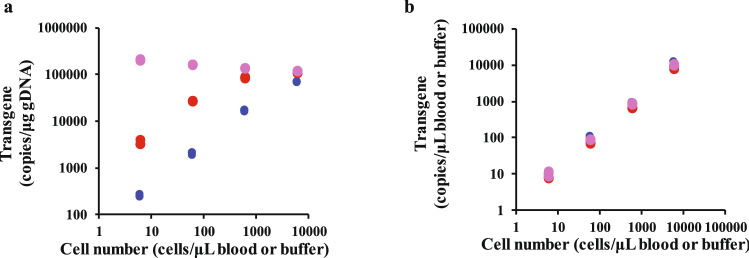
Ex vivo dose-proportionality of transgene copy numbers. Each point shows the transgene level expressed in units of (**a**) copies/μg gDNA and (**b**) copies/μL blood or buffer against cell concentration in human blood or buffer. Blue, red, and pink points represent healthy human blood, lymphocyte-depleted human blood, and buffer, respectively. Target1-CAR T-cells were added to healthy human blood, lymphocyte-depleted human blood, and buffer at final concentrations of 3000 to 300,000 cells/0.5 mL blood or buffer (600 to 60,000 cells/μL blood or buffer). The samples were prepared in triplicate for each concentration.

Using the validated method, we evaluated the dose–response relationship of transgene copy number against CAR T-cell number (Fig. 3). The target1-CAR T cells were added to human blood from healthy volunteers, lymphocyte-depleted human blood, and buffer without any gDNA at a concentration range of 3000 to 300,000 cells/0.5 mL blood or buffer (600 to 60,000 cells/μL blood or buffer). We found that transgene increase expressed in units of copies/μg gDNA was saturable with increasing CAR T cells in lymphocyte-depleted blood samples, whereas the transgene level increased in a dose-proportionate manner in blood from healthy humans. Notably, the transgene level did not increase in the buffer when the results were expressed in units of copies/μg gDNA. This is likely due to the simultaneous increase in CAR transgenes and gDNA derived from CAR T cells. In contrast, a clear dose-proportionality of the transgene level was observed when the results were expressed in units of copies/μL blood, and each observed value was identical in all samples. These results indicate that the transgene level expressed in units of copies/μg gDNA is highly affected by variations in background gDNA levels, whereas our validated method enables accurate transgene concentration quantification and evaluation of CAR T-cell expansion without underestimation.

### Evaluation of in vivo expansion

**Figure 4:**
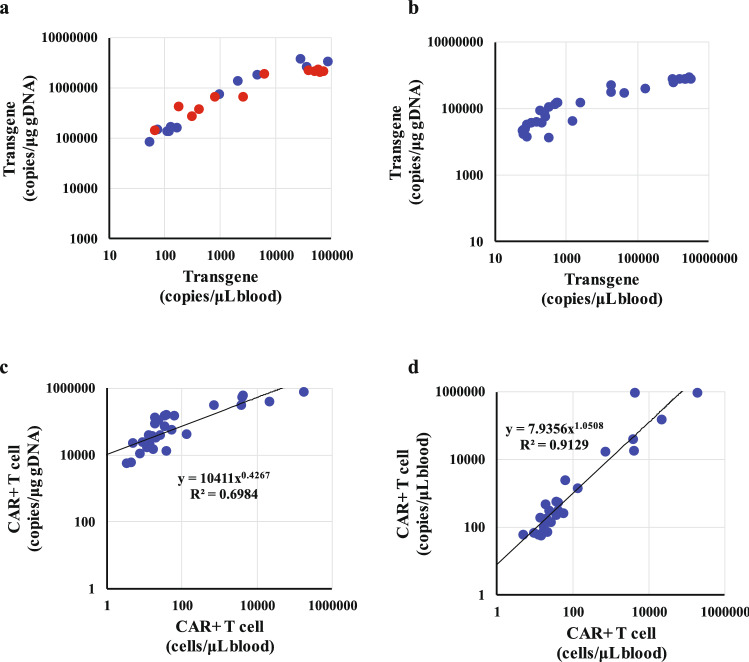
In vivo expansion of target1- and target2-CAR T-cells after injection in immunodeficient mice (n = 3 to 5 per dosage). Each point shows transgene level or CAR T-cell number. (**a**) Comparison of target1-CAR T-cell level expressed in units of copies/μg gDNA and copies/μL blood. Blue and red points represent doses of 5 × 10^6^ and 10 × 10^6^ CAR+ cells/animal, respectively. (**b**) Comparison of target2-CAR T-cell level expressed in units of copies/μg gDNA and copies/μL blood. (**c**) Comparison of target2-CAR T-cell level expressed in units of copies/μg gDNA and CAR+ cell number quantified by flow cytometry. (**d**) Comparison of target2-CAR T-cell level expressed in units of copies/μL blood and CAR+ cell number quantified by flow cytometry.

We quantified the in vivo expansion of two types of CAR T-cell with different single-chain variable fragments (target1- and target2-CAR T cells) after administration to cell line-derived xenograft mice in units of copies/μg gDNA and copies/μL blood. Target1-CAR T cells were injected at doses of 5 × 10^6^ and 10 × 10^6^ CAR+ cells/animal (CAR-positive rate is 47% to 52%). A comparison of the two units revealed that the transgene level in units of copies/μg gDNA was saturated at more than 1000 copies/μL blood and the expansion range expressed in units of copies/μL blood was an order of magnitude greater than that expressed in units of copies/μg gDNA (Fig. 4a). A similar phenomenon was observed in the study of target2-CAR T cells (Fig. 4b). These results are consistent with ex vivo dose-proportionality data (Fig. 3). Furthermore, we compared the data from qPCR and flow cytometry. The correlation between target2-CAR T cell number and transgene level was much more apparent when expressed in units of copies/μL blood than when expressed in units of copies/μg gDNA (Fig. 4c,d). Expression of the transgene level in units of copies/μg gDNA showed saturation at more than 100 cells/μL blood, while units of copies/μL blood showed strong correlation according to the linear regression analysis (r^2^ = 0.9129). These results suggest that the unit notation of copies/μg gDNA carries the risk of underestimating the in vivo expansion, whereas copies/μL blood is a more appropriate unit for the evaluation of cellular kinetics.

## Discussion

We demonstrate herein a new qPCR methodology for the determination of CAR T-cell kinetics as expressed in volume-based units. This methodology yields acceptable quantitative results in terms of selectivity, linearity, accuracy, and precision. In comparison to the conventional approach, our methodology enables greater accuracy for evaluating in vivo CAR T expansion, while avoiding underestimation. Our results indicate that the unit of copies/μL blood is more appropriate for the evaluation of cellular kinetics than the unit of copies/μg gDNA, as the latter is highly affected by variations in background gDNA levels.

The unique kinetic profile of CAR T cells has been demonstrated through the conventional method, with an initial transient decline a few days after administration, followed by rapid expansion^4–6^. However, based on the results of the present study, we expect that these profiles might be erroneous. Patients generally receive chemotherapy to deplete lymphocytes before CAR T administration^16,17^, which leads to a dramatic decrease in blood gDNA levels^8,9^. This transient decrease in gDNA likely causes overestimation of copy number as expressed in units of copies/μg gDNA during this time. Conversely, the subsequent recovery of gDNA by lymphocyte repopulation would lead to a decrease in the enumerated copies/μg gDNA. Additionally, the CAR T-cell expansion that follows lymphocyte repopulation would result in an increase in measured copies/μg gDNA, until CAR T-cell-derived gDNA predominates relative to the background gDNA level from endogenous WBCs^4,18,19^. Furthermore, expression of peak expansion in units of copies/μg gDNA may lead to underestimation in a predominantly CAR-positive population because both CAR transgenes and CAR T-cell-derived gDNA would increase simultaneously, as shown in Figs. 3 and 4. It has been reported that the dose-dependent peak of blood expansion does not occur at a dose of more than 50 × 10^6^ CAR T-cells^20^. As our methodology expresses cellular kinetics in units of copies/μL blood, it eliminates the influence of gDNA variability; thus, “true cellular kinetics” are revealed using copies/μL blood as the unit of notation for cellular kinetic studies.

An early indicator is needed to assess the clinical response in patients undergoing CAR T therapy^21^. Several studies have indicated that patients who demonstrate a positive response show long-term persistence of CAR T cells^3,5^. However, persistence is not a useful predictor of clinical response, as it is a long-term evaluation metric, whereas clinical response requires an assessment near the time of treatment. An alternative predictor could be in vivo peak expansion. A significant difference has been reported between the peak expansions of responsive and non-responsive patients^20,22,23^. However, the peak expansion distribution overlapped between these two groups, and therefore the peak expansion in responsive patients did not differ greatly from that in partially responsive patients. This indicates that peak expansion is a promising predictor, but not the perfect one. The conventional method carries the risk of underestimating peak expansion, particularly during the initial proliferation of CAR T cells, and when the CAR-positive population becomes predominant. If that happens, our methodology may be useful to distinguish responsive patients from partially and non-responsive patients at an early stage, and thereby provide another therapeutic opportunity for patients in need. To verify the potential usefulness of our methodology, it is necessary to perform cell quantification by two methods and compare CK/efficacy in clinical trials.

According to the guidance provided by the Food and Drug Administration entitled “Long Term Follow-Up After Administration of Human Gene Therapy Products,” PCR assays should have a demonstrated limit of quantitation (LoQ) of fewer than 50 copies/μg gDNA for the detection of transgenes after administration of gene therapy products^24^. With the exception of adoptive immunotherapy with gene-modified cell products, the conventional notation is appropriate for verifying transgene persistence, as such gene therapy products would not substantially alter the background gDNA content in biological samples. However, in CAR T-cell therapy, the background gDNA varies highly under conditions of chemotherapy and CAR T proliferation, which causes fluctuations in assay sensitivity; the LoQ value (copies/μg gDNA) of a sample with a high gDNA level would be lower than that of a sample with low gDNA content, even if the copy number per unit of volume is constant. Additionally, blood samples collected immediately after chemotherapy might be excluded from PCR analysis under the conventional approach due to a low yield of DNA. Our methodology overcomes these issues through its applicability to various types of blood samples, including lymphodepleted blood.

From the perspective of pharmacokinetic analysis, it is important to evaluate the biodistribution of the analyte by comparing concentrations between blood and tissue samples^25,26^. However, direct comparison in units of copies/μg gDNA is difficult^27^, because gDNA content varies according to tissue type^28^. However, the volume-based unit is applicable to the quantification of CAR transgenes in tissue samples, as this unit enables direct comparison of concentrations between tissues and facilitates comprehensive analysis such as physiologically based pharmacokinetic (PBPK) modeling^29^. Further study is needed to clarify whether our method can be applicable to tissue samples.

In conclusion, the present study elucidated the usefulness of a newly developed qPCR method for the evaluation of CAR T-cell kinetics. We propose the use of the volume-based unit (copies/μL blood) for cellular kinetics studies in CAR T-cell therapy.

## Methods

### Materials

Plasmids for target1-CAR and target2-CAR genes were prepared by Takara Bio Inc. (Shiga, Japan) and GENEWIZ (South Plainfield, NJ, USA), respectively. Dog genomic DNA was purchased from Zyagen (San Diego, CA, USA). Human and mouse gDNA was purchased from Thermo Fisher Scientific (Waltham, MA, USA). Mouse blood was obtained from immunodeficient mice (NOD.Cg-PrkdcscidIl2rgtm1Wjl/SzJ, female, 8-week old; Charles River Laboratories Japan, Inc., Kanagawa, Japan). The animal protocol was approved by the Institutional Animal Care and Use Committee of the Shonan Health Innovation Park (Kanagawa, Japan, Approval No. AU-00020703). Human blood was obtained from eight healthy volunteers with written informed consent. The procedure was approved by the ethics committee of Takeda Pharmaceutical Company Limited (Osaka, Japan). All methods were performed in accordance with relevant guidelines and regulations.

### Primers and probes

Primers and probes for CAR transgenes and dog MC1R were synthesized by Thermo Fisher Scientific as follow: F-primer for target1-CAR: 5′-GGAGCTGAGGTCCCTGAGAAG-3′, R-primer for target1-CAR: 5′-CCTGGCCCCAGTAGTCGAA-3′, probe for target1-CAR: 5′-CGACTACAGGGCCTACT-3′, F-primer for target2-CAR: 5′-CCGGAGTGCTGCTGATCAG-3′, R-primer for target2-CAR: 5′-GCTGTGCCAGATCATGCAGTA-3′, probe for target2-CAR: 5′-CTGAGGAGCGAGGATG-3′, F-primer for dog MC1R: 5′-CGCCCATGTATTACTTCATCTGTTGCC-3′, R-primer for dog MC1R: 5′-CACGGCGATGGCGCCCAGGAA-3′, probe for dog MC1R: 5′-GCCTTGGCTGCGCAGGCTGCTGTGGTGCAG-3′. The 5′ end of the probe was conjugated with the fluorescence dye FAM, and the 3′ end was conjugated with non-fluorescent quencher and minor groove binder. Primer or probe sequences for dog MC1R were previously identified^30^. For the quantification of human and mouse gDNA, TaqMan RNase P Control Reagents Kit and TaqMan Copy Number Reference Assay, mouse, Tfrc (Thermo Fisher Scientific) were used, respectively.

### Development of qPCR method for volume-based unit

The plasmid carrying the target1-CAR transgene was diluted to 2 × 10^8^ copies/µL with AE buffer (Qiagen) in a 1.5 mL DNA LoBind tube (Eppendorf, Hamburg, Germany). The solution was diluted with AE buffer to final concentrations of 1.5 × 10, 2.5 × 10, 5 × 10, 1.5 × 10^2^, 5 × 10^2^, 1.5 × 10^3^, 5 × 10^3^, 5 × 10^4^, 5 × 10^5^, and 5 × 10^6^ copies/µL (standard solution). Pooled blank blood from healthy volunteers (1.8 mL) was dispensed into a 15 mL DNA LoBind tube (Eppendorf), and then the standard solutions were added to produce final concentrations of 0.75, 1.3, 2.5, 7.5, 2.5 × 10, 7.5 × 10, 2.5 × 10^2^, 2.5 × 10^3^, 2.5 × 10^4^, and 2.5 × 10^5^ copies/µL blood. Two aliquots of each concentration of standard samples for the calibration curve were prepared. QC samples with concentrations of 1.1, 3.3, 55.6, and 5555.6 copies/µL blood were prepared using pooled blank blood from healthy volunteers, as well as standard samples for the calibration curve. Four aliquots of each of the QC sample concentrations were prepared. Dog gDNA (1 ng) was added to all samples for experiments using an external control gene. DNA from blood samples was extracted using a QIAamp DNA Midi Kit (Qiagen) according to the manufacturer’s protocol. The mixture containing QIAGEN Proteinase (200 μL), Buffer AL (2.4 mL), and blood samples was incubated at 70 °C for 30 min. Ethanol (2 mL; FUJIFILM Wako Pure Chemical Corporation, Osaka, Japan) was added and mixed for 30 s. The mixture was transferred to the QIAamp Mini Spin Column and was centrifuged at 220×*g* for 3 min and then at 1850×*g* for 3 min. Buffer AW1 (2 mL) was added, and the mixture was centrifuged at 4420×*g* for 3 min. Buffer AW2 (2 mL) was added, and the mixture was centrifuged at 4420×*g* for 15 min. DNA was extracted using 200 μL of Buffer AE. Total volume for the PCR was 40 μL, containing 15 μL of DNA template; 20 μL of TaqPath ProAmp Master Mix (Thermo Fisher Scientific); and 5 μL of the mixture of forward primer, reverse primer, TaqMan probe, and water. A duplex assay was used to simultaneously detect the CAR transgene and either the internal control (human *RNaseP*) or external control (dog *MC1R*) genes. The final concentrations of the primers and probe for the CAR transgene were 150 nmol/L and 250 nmol/L, respectively. The TaqMan RNase P Control Reagents Kit was used to detect the internal control. The final concentration of the primers and probe for the external control gene was 500 nmol/L. QuantStudio 7 Flex Real Time PCR System with SDS software (Thermo Fisher Scientific) was used for qPCR. The qPCR program was set as follows: 95 °C for 10 min, and 40 cycles at 95 °C for 15 s/ 60 °C for 60 s. Reactions for each sample were performed in duplicate, and the mean values of Ct were used for calculating the copy number. The calibration curve was constructed by linear regression analysis using the following equation: X = 10 (Y − b)/a, where "X" is the nominal concentration (copies/μL blood), "Y" is the Ct in PCR from each sample, and "a" and "b" are the slope and the y-intercept, respectively. The PCR efficiency was calculated by the formula E = 10^(−1/slope)^ – 1. The accuracy (%) and CV (%) of the observed values were calculated to evaluate the validity of the method.

### Method validation for quantification of human blood samples

Method validation was conducted as described in “Development of qPCR method for volume-based unit” section, with minor modifications, on three separate days. As blood sampling was limited due to patient safety concerns, blood volume was reduced to 500 μL. Pooled blank blood from healthy volunteers (0.5 mL) was dispensed into 5 mL DNA LoBind tubes (Eppendorf), and then the standard solutions were added to produce final concentrations of 0.75, 1.3, 2.5, 7.5, 2.5 × 10, 7.5 × 10, 2.5 × 10^2^, 2.5 × 10^3^, 2.5 × 10^4^, and 2.5 × 10^5^ copies/µL blood. Two aliquots of each concentration of the standard sample were prepared for the calibration curve. QC samples were prepared using pooled blank blood at concentrations of 7.5, 2.0 × 10^2^, and 2.0 × 10^5^ copies/µL blood; four aliquots of each concentration were prepared. Dog gDNA (1 μg) was added to all samples used for methods with the external control gene. DNA extraction, PCR, and copy number calculation were conducted as described in “Development of qPCR method for volume-based unit” section. All PCRs were performed in triplicate per individual sample. The relative errors (RE [%]) and CV of observed values were calculated to evaluate the validity of the method.

### Method validation for quantification of mouse blood samples

Three validation batches were processed on three separate days. The plasmid carrying the target CAR transgene was diluted to 2 × 10^8^ copies/µL with AE buffer (Qiagen) in a 1.5 mL DNA LoBind tube. The standard samples for the calibration curve were prepared from this solution with final concentrations of 50, 2.0 × 10^2^, 6.0 × 10^2^, 2.0 × 10^3^, 6.0 × 10^3^, 2.0 × 10^4^, 6.0 × 10^4^, 2.0 × 10^5^, and 2.5 × 10^6^ copies/µL blood (blood volume, 30 μL). Two aliquots of each concentration were prepared. QC samples with concentrations of 50, 1.0 × 10^2^, 2.0 × 10^2^, 6.0 × 10^2^, 4.0 × 10^3^, 4.0 × 10^4^, 4.0 × 10^5^, and 2.0 × 10^6^ copies/µL blood were prepared using pooled blank blood; five aliquots of each concentration were prepared. DNA from a blood sample was extracted using a QIAamp DNA Micro Kit (Qiagen) according to the manufacturer’s protocol. ATL buffer (80 μL), Proteinase K (16 μL), and dog gDNA (1.2 ng) were added to blood samples. Buffer AL (100 μL) was added to the mixture of ATL buffer and blood sample, and then incubated at 56 °C for 30 min. Ethanol (50 μL) was added and mixed for 30 s, and then the mixture was transferred to the QIAamp Micro Spin Column and was centrifuged at 6000×*g* for 15 min. Buffer AW1 (0.5 mL) was added and the mixture was centrifuged at 6000×*g* for 2 min, followed by the addition of Buffer AW2 (0.5 mL) and centrifugation at 6000×*g* for 2 min. To dry the membrane completely, the spin column was centrifuged at 20,000×*g* for 3 min. DNA was extracted using 100 μL of Buffer AE. The PCR for the detection of CAR transgenes and dog *MC1R* was performed as described in “Development of qPCR method for volume-based unit” section. All reactions were performed in duplicate per individual sample.

### In vitro dose-dependent study

To prepare lymphodepleted human blood, blood from healthy volunteers was passed through a Plasmodipur filter (EuroProxima B.V., Arnhem, Netherlands) four times. Target1-CAR T cells were prepared using peripheral blood mononuclear cells from healthy donors. Isolated T cells were cultured and activated by interleukin-2. The CAR gene was transduced into activated T-cells using a retroviral vector (Takara Bio Inc.), and then the transduced T cells were expanded. The CAR-positive rate was confirmed as 31.1% by flow cytometry. The cells were counted using the FACSLyric flow cytometer (BD Biosciences, CA, USA) and diluted to concentrations of 6, 60, 600, and 6000 cells/µL blood or buffer using pooled human blood, lymphodepleted blood, and CELLOTION (a buffer, Zenoaq resource Co., Ltd, Fukushima, Japan). CAR transgenes were quantified in all samples with the validated spike-in method and conventional method. For the conventional method, the concentrations of CAR transgenes and gDNA in the eluate were evaluated using a standard solution for the calibration curve. The concentrations of standard solution for the CAR transgene were 1.5, 5, 1.5 × 10, 2.5 × 10, 5.0 × 10, 1.5 × 10^2^, 5.0 × 10^2^, 1.5 × 10^3^, 5.0 × 10^3^, 1.5 × 10^4^, 5.0 × 10^4^, 1.5 × 10^5^, and 5.0 × 10^5^ copies/µL AE buffer. The concentrations of standard solution for human gDNA were 5.6 × 10^–3^, 5.6 × 10^–2^, 5.6 × 10^–1^, 5.6, 5.6 × 10, 1.1 × 10^2^, and 2.2 × 10^2^ ng/µL AE buffer. PCRs were carried out for both the conventional method and the spike-in method. The CAR transgene level was described as copies/μL blood and copies/μg gDNA.

### In vivo study

Three cell line-derived xenograft mice (NOD.Cg-PrkdcscidIl2rgtm1Wjl/SzJ) were used for each group. Target1- and target2-CAR T cells were prepared as described in “In vitro dose-dependent study” section. The CAR-positive rate was confirmed as 47% to 52% by flow cytometry. Target1-CAR T cells were intravenously administrated at doses of 5 × 10^6^ and 10 × 10^6^ CAR+ cells/animal. At each time point (1, 3, 7, 10, 14, 17, 21, and 28 days after a single intravenous administration), mouse blood was collected from the submandibular vein and stored at – 80 °C. The CAR-positive rate for target2-CAR T cells was confirmed as 28.0 to 69.1% by flow cytometry. Target2-CAR T-cells were intravenously administrated at a dose of 5 × 10^6^ CAR+ cells/animal. Mouse blood was collected at each time point (1, 4, 8, 11, 15, 22, and 29 days after a single intravenous administration) from the submandibular vein and was used to determine the absolute CD3+ cell count and assess the CAR-positive rate via flow cytometry. Additional blood was collected at each time point, except for the endpoint, and pooled for the qPCR assay. For the qPCR assay, blood samples were stored at – 80 °C. For the absolute CD3+ cell count and CAR-positivity assessment, fresh blood samples were used; CD3+ cell counting was conducted as previously reported^31^. The blood sample (10 μL) was mixed with Hank's Balanced Salt Solution (40 μL, Thermo Fisher Scientific), anti-CD3 (FITC), and anti-CD45 (PerCP-cy5) in BD Trucount tubes (BD Biosciences, CA, USA). After incubation for 15 min in the dark at approximately 25 °C, 450 μL of tenfold diluted BD FACS lysing solution containing formaldehyde (BD Biosciences) was added to lyse the red blood cells. Samples stained with the lyse-no-wash procedure were transferred to the FACSLyric flow cytometer. The CD3/45+ population was identified using FlowJo software (version 10, BD Biosciences) and the cells were counted. For CAR+ phenotyping, the Zombie NIR Fixable Viability Kit (BioLegend, CA, USA), anti-CD3 (FITC), His-tagged target2 substrate, and anti-His-tag (PE) reagents were used. The blood samples (30 μL) were stained using a lyse/wash procedure and transferred to a FACSLyric flow cytometer. The live/CD3/CAR+ population was identified using FlowJo software. The absolute number of CAR+ cells was calculated by the following equation: CD3+ cell number × CAR + population among CD3+ cells. The CAR transgenes in blood samples (30 μL) were quantified with the validated method as described in “Method validation for quantification of mouse blood samples” section. For the quantification of mouse gDNA, the TaqMan Copy Number Reference Assay, mouse, Tfrc was used. For the calculation of copies/μg gDNA, the sum of human and mouse gDNA amount in the eluate was used.
